# Interleukin-6 Treatment Results in GLUT4 Translocation and AMPK Phosphorylation in Neuronal SH-SY5Y Cells

**DOI:** 10.3390/cells9051114

**Published:** 2020-04-30

**Authors:** Daniel M. Marko, Gregory Foran, Filip Vlavcheski, David C. Baron, Grant C. Hayward, Bradley J. Baranowski, Aleksander Necakov, Evangelia Tsiani, Rebecca E. K. MacPherson

**Affiliations:** 1 Department of Health Sciences, Brock University, St. Catharines, ON L2S 3A1, Canada; dm14vw@brocku.ca (D.M.M.); fv11vi@brocku.ca (F.V.); dxbaron09@gmail.com (D.C.B.); ghayw038@ottawau.ca (G.C.H.); bb12qf@brocku.ca (B.J.B.); etsiani@brocku.ca (E.T.); 2 Department of Biological Sciences, Brock University, St. Catharines, ON L2S 3A1, Canada; gf12ks@brocku.ca (G.F.); anecakov@brocku.ca (A.N.); 3 Centre for Neuroscience, Brock University, St. Catharines, ON L2S 3A1, Canada; 4 Centre for Bone and Muscle Health, Brock University, St. Catharines, ON L2S 3A1, Canada

**Keywords:** interleukin-6, insulin, SH-SY5Y Cells, AMPK, Akt, glucose uptake, Alzheimer’s disease

## Abstract

Interleukin-6 (IL-6) is a pleiotropic cytokine that can be released from the brain during prolonged exercise. In peripheral tissues, exercise induced IL-6 can result in GLUT4 translocation and increased glucose uptake through AMPK activation. GLUT4 is expressed in the brain and can be recruited to axonal plasma membranes with neuronal activity through AMPK activation. The aim of this study is to examine if IL-6 treatment: (1) results in AMPK activation in neuronal cells, (2) increases the activation of proteins involved in GLUT4 translocation, and (3) increases neuronal glucose uptake. Retinoic acid was used to differentiate SH-SY5Y neuronal cells. Treatment with 100 nM of insulin increased the phosphorylation of Akt and AS160 (*p* < 0.05). Treatment with 20 ng/mL of IL-6 resulted in the phosphorylation of STAT3 at Tyr705 (*p* ≤ 0.05) as well as AS160 (*p* < 0.05). Fluorescent Glut4GFP imaging revealed treatment with 20ng/mL of IL-6 resulted in a significant mobilization towards the plasma membrane after 5 min until 30 min. There was no difference in GLUT4 mobilization between the insulin and IL-6 treated groups. Importantly, IL-6 treatment increased glucose uptake. Our findings demonstrate that IL-6 and insulin can phosphorylate AS160 via different signaling pathways (AMPK and PI3K/Akt, respectively) and promote GLUT4 translocation towards the neuronal plasma membrane, resulting in increased neuronal glucose uptake in SH-SY5Y cells.

## 1. Introduction

The human brain represents only 2–3% of total body mass, however it accounts for ~20–30% of the whole-body energy expenditure. This energy is mostly derived from glucose, of which the brain utilizes ~100–150 g per day [1,2]. The transport of glucose into the brain and into neurons is an important regulatory site of glucose metabolism and it is now well established that a reduction in glucose uptake and metabolism is observed in various neurodegenerative diseases, such as Alzheimer’s disease (AD) [3,4,5,6]. In fact, the use of positron emission tomography (PET) to analyze cerebral glucose utilization has become one of the most reliable tools for diagnosing and assessing the progression of AD [7]. For example, longitudinal 18F-FDG PET studies in healthy individuals who later developed AD demonstrated that metabolic reductions precede the onset of clinical symptoms by several years and correlate with the diagnosis and severity of AD [8]. Given this information, the deregulation of glucose uptake and utilization in AD has become an important therapeutic target.

The brain expresses several members of the glucose transporter family (GLUT), which function to increase glucose uptake into cells, including neurons. GLUT1 and GLUT3 are the major glucose transporters that are found in the brain and facilitate glucose transport across the cell membrane independently of insulin [9,10,11]. GLUT1 is ubiquitously expressed in the brain, but the majority is found in endothelial cells of the microvasculature and astrocytes, whereas GLUT3 is the canonical neuronal glucose transporter [9,11,12,13,14]. The observed lower glucose utilization in patients with AD as well as animal models of AD has been associated with a decreased content of GLUT1 and GLUT3 in the cortex and hippocampus which are major structures associated with cognition and memory [15,16,17,18].

Interestingly, neurons also express insulin sensitive GLUT4, providing another means by which neurons may take up glucose. GLUT4 is expressed in several brain regions but the largest expression levels are found within the cortex and hippocampus of the brain [19,20]. Unlike the other GLUT isoforms, GLUT4 levels can be regulated by both, the insulin (PI3K/Akt) and AMP-activated protein kinase (AMPK) pathways, which promote translocation of GLUT4 to the plasma membrane to increase cellular glucose uptake [19,21,22,23,24]. In metabolically healthy individuals, an acute elevation of insulin can have a beneficial impact on neuronal metabolism and glucose uptake, as well as cognitive function, specifically through the binding of insulin to its membrane bound receptor, the downstream activation of Akt, and the resultant activation and translocation of GLUT4 [25]. Conversely, when insulin levels are chronically elevated, as is the case in metabolically unhealthy individuals, neuronal insulin resistance can develop [26,27], which may result in reduced insulin-mediated GLUT4 translocation and neuronal glucose uptake [25]. This phenomenon is similar to that observed in the periphery with insulin resistance and type 2 diabetes, and has led some researchers to consider AD as a form of diabetes termed type 3 diabetes [27,28]. This highlights the need to find insulin independent mechanisms to increase neuronal glucose uptake.

It well known that exercise increases glucose uptake in skeletal muscle in an insulin independent manner through contraction-driven activation of AMPK and translocation of GLUT4 transporters into the plasma membrane [29,30,31,32]. Exercise is also known to increase brain glucose uptake and use, however the underlying mechanisms stimulating the increased glucose uptake remain unknown [33]. Interleukin-6 (IL-6) is a pleiotropic cytokine released in response to exercise from several tissues including the brain, however the role of acute brain-derived IL-6 still remains elusive [34,35,36]. IL-6 mediates its effect via two mechanisms, trans-signaling and classical signaling. With trans-signaling, IL-6 binds to a soluble IL-6 receptor (sIL-6R), which is then able to bind to and activate gp130 homodimers on cells that do not express membrane-bound IL-6R, thereby mediating ‘trans-signaling’ and increasing the potential range of IL-6 target tissues and activities [37]. IL-6 trans-signaling plays an important role in pathological signaling in the brain [38], however classical IL-6 signaling may play an important physiological role in neurons. In classical IL-6 signaling, IL-6 first binds to a membrane-bound IL-6 receptor (IL-6R) that associates with a homodimer of gp130 which transmits the intracellular signal. IL-6 typically signals through the gp130 receptor, with the Janus kinase/signal transducer and activator of transcription (JAK/STAT) pathway being the major intracellular mediator of IL-6 effects [39]. All components of the classical IL-6 signaling pathway (IL-6, IL-6R, and the signal-transducing component gp130) are detectable in the brain, with evidence of altered cortical immunoreactivity of the functional IL-6 receptor complex in AD [40]. It has further been demonstrated that neurons express IL-6R mRNA and gp130 protein [41,42,43], and that although both neurons and glia release IL-6, only neurons show detectable levels of IL-6R and the related gp130 subunit required for active signalling [44]. Within peripheral tissues, such as skeletal muscle and adipose tissue, the current understanding is that acute IL-6 exposure results in STAT3 phosphorylation and subsequent AMPK activation, which increases GLUT4 translocation to the plasma membrane [45,46,47,48,49,50].

Activation of AMPK is accomplished via the phosphorylation of its Thr172 residue, and once activated, AMPK can phosphorylate Akt substrate of 160 kDa (AS160), which directly interacts with the GLUT4 storage vesicles (GSVs) [45,46,47,48]. The interaction of phosphorylated AS160 and the GSVs promotes GLUT4 translocation to the plasma membrane to allow for glucose uptake into the cell [45,46,47,48]. It remains unknown if this IL-6 induced cascade of events occurs in neurons similarly to that observed in skeletal muscle. There have been several studies showing that IL-6 is directly released from the brain during exercise [34,35,36], however there have been no studies examining if IL-6 has a role on neuronal glucose uptake.

The purpose of this study was three pronged: the first objective was to determine if acute IL-6 exposure could activate AMPK in SH-SY5Y neuronal cells, the second objective was to determine if proteins involved in GLUT4 translocation were activated with acute IL-6 exposure, with the overall goal of investigating whether IL-6 can promote GLUT4 translocation, and finally the third objective was to determine if IL-6 exposure increased cellular glucose uptake. This study will be the first to examine the effects of IL-6 on neuronal GLUT4 translocation and the first to investigate the mechanisms that are involved. This information significantly increases our fundamental understanding of the processes that underlie how brain glucose uptake and metabolism are regulated.

## 2. Materials and Methods

### 2.1. Materials

High glucose Dulbecco’s Modified Eagle’s medium (DMEM) (cat# D6429-500 mL), non-essential amino acid solution (cat# M7145-100 mL), penicillin/streptomycin solution (cat# P4333-100 mL), trypsin EDTA solution (cat# T4049-500 mL), fetal bovine serum (FBS) (cat# F1051-500 mL), Retinoic acid (cat# R2625), cytochalasin B (cat# C6762-5MG), and retinoic acid (cat# R2625) were acquired from Sigma-Aldrich (Oakville, ON, Canada). Dimethyl sulfoxide (DMSO) was acquired from ACP Chemicals (cat# D6400-1L, Saint-Leonard, QC, Canada). SH-SY5Y cells used for cell culture were donated by Dr. J. Stuart from Brock University. Cell culture plates (6-well) were from Fisher Scientific (cat# 10062-892; Walham, MA, USA) and 35mm glass bottom collagen coated dishes for fluorescent imaging were from MatTek Life Sciences (cat# P35GCOL-1.5-14-C, Ashland, MA, USA). Insulin (Humulin) was purchased from Eli Lilly. Human IL-6 was obtained from PeproTech (cat# 200-06-2046, Rocky Hill, NJ, USA). NP40 Cell Lysis Buffer was purchased from Life Technologies - ThermFiher (cat# FNN0021, Walham, MA, USA) and supplemented with phenylmethylsulfonyl fluoride and protease inhibitor that was purchased from Sigma-Aldrich (cat# 7626-5G, cat# P274-1BIL, Oakville, ON, Canada). Antibodies against STAT3 (1:500, Cell Signaling, cat# 8768), pSTAT3 Tyr705 (1:500, Cell Signaling, cat# 9138), AMPK (1:500, Cell Signaling, cat# 2793S), pAMPK Thr172 (1:500, Cell Signaling, cat# 25315), ACC (1:500, Cell Signaling, cat# 3676), pACC Ser 79 (1:500, Cell Signaling, cat# 3661), AS160 (1:500, Millipore, cat# 1604977), pAS160 (1:500, Invitrogen, cat# 44-1071), Akt (1:500, Cell Signaling, cat# 4685S), pAkt Ser473 (1:500, Cell Signaling, cat# 4058S) and GAPDH (1:1000, Cell Signaling cat#2118) were used for this study (Cell Signaling Technology, Danvers, MA, USA),. Horseradish peroxidase-conjugated donkey anti-rabbit (cat# 711-035-152) and goat anti-mouse (cat# 115-035-003) IgG secondary antibodies were from Jackson ImmunoResearch Laboratories (Westgrove, PA, USA). Molecular weight marker, reagents, and nitrocellulose membranes for SDS-PAGE were acquired from Bio-Rad (Mississauga, ON, Canada) and GE Healthcare Life Science (cat# 10600002). Western lightning Plus-ECL (cat# 105001EA) and [3H]-2-deoxy-D-glucose (cat# NET549001MC) were obtained from PerkinElmer (Waltham, MA, USA).

### 2.2. Cell Culture

Undifferentiated SH-SY5Y cells at passage 14 were cultured in DMEM containing 10% FBS, 2% non-essential amino acids (NEAA), and 1% penicillin/streptomycin. The use of this cell line was approved by the Research Ethics Board at Brock Univesity (#17-397). Cells were left to grow until 80–90% confluent in a flask. Cells were then seeded into 6-well plates and cultured in DMEM supplemented with 1% FBS, 2% NEAA, and 1% penicillin/streptomycin, along with 10 μM of retinoic acid to initiate the differentiation process [51]. Differentiation was done for 5 days with the media and retinoic acid being changed every two days. Media were then changed to high glucose DMEM supplemented with 0.1% FBS, 2% NEAA, and 1% penicillin/streptomycin prior to the experiments where cells were treated acutely for 30 min with 100 nM of insulin, 10 ng/mL of IL-6, or 20 ng/mL of IL-6. To evaluate the time course response of IL-6 on AMPK phosphorylation cells were treated with 20 ng/mL of IL-6 for 10, 20, 30, and 60 min. Following treatment, cells were then lysed using 100 μL of NP40 Cell Lysis Buffer supplemented with 34 μL phenylmethylsulfonyl fluoride and 50 μL protease inhibitor cocktail. Lastly, the cells were scraped and collected in tubes where they were sonicated for two 20-s bouts on ice and stored in a −80 °C freezer for future analysis. For fluorescent image analysis, cells were seeded on 35 mm Matek glass bottom collagen coated dishes. Cell culture, differentiation, and stimulation with insulin or IL-6 was performed as indicated above [51].

### 2.3. Cell Transfection

SH-SY5Y cells were serum deprived for 1 h prior to transfection by exposure to high glucose DMEM media lacking any FBS. GLUT4myc-GFP DNA constructs were received from Drs. A. Klip and P. Bilan at The University of Toronto and cell transfection was adapted from previously published work [52]. Lipofectamine 3000 (Thermo Scientific, cat# L3000015) was used for all transfections using a 6:4:2 ratio of lipofectamine 3000:P3000 reagent: micrograms of DNA per plate. Following the mixing of all components together as per the Lipofectamine 3000 protocol, transfection solution was incubated at room temperature for 20 min. Transfection mixture was then added to cells drop-by-drop and allowed to incubate on the cells for 6 h at 37 °C and 18.5% O_2_. Following the 6-hour incubation, cells had their media changed to high glucose DMEM containing 1% FBS, 2% NEAA, and 1% penicillin/streptomycin. Cells were then imaged between 30- and 36-h post-transfection.

### 2.4. Western Blotting

Protein concentrations in the cell lysates were determined using a bicinchroninic acid (BCA) quantification assay [53]. Equal amount of protein was loaded on polyacrylamide gels and separated by electrophoresis. Proteins from the gel were wet transferred onto nitrocellulose membranes for 1 h at 100 V on ice. The membranes were subsequently placed in a 5% dry milk-Tris-buffered saline/0.1% Tween 20 (TBST) blocking buffer for 1 h. Primary antibody was prepared at a 1:500 ratio in a 5% bovine serum albumin (BSA) and TBST solution, and membranes were incubated overnight on a rocker at 4 °C. Following the overnight incubation, the membranes were washed 3 × 5 min with TBST. The appropriate horseradish peroxidase secondary antibody was prepared in a 1:2000 1% milk solution before being added to the membranes, which incubated at room temperature for 1.5 h. Signals were detected using enhanced chemiluminesence and were subsequently quantified by densitometry using a Bio-Rad ChemiDoc imaging system. A representative Ponceau stain was analyzed in addition to a protein loading control (GAPDH) for each membrane to ensure equal loading (<10% variability across the membrane) [54]. Western blot analysis was used to examine protein content of proteins involved in the IL-6 signaling cascade, namely STAT3, pSTAT3, AMPK, pAMPK, ACC, pACC, AS160 and pAS160. Furthermore, proteins involved in the insulin cascade were also investigated, namely Akt and pAkt.

### 2.5. Microscopy

All imaging was completed with a Carl Zeiss Axio Observer. Z1 inverted light/epifluorescence microscope equipped with ApoTome.2 optical sectioning and a Hamamatsu ORCA-Flash 4.0 V2 digital camera. Cells were viewed with a Plan-Apochromat 63×/1.40 Oil DIC M27 microscope objective. Throughout the experiments, cells were kept in a humidified incubator with 5% CO_2_ at 37 °C. Green fluorescence was detected using a fluorescence channel possessing excitation and emission wavelength filter sets of 450–490 nm and 500–550 nm, respectively. Both the intensity of fluorescence illumination and camera exposure time were held constant throughout all experiments. For time-lapse experiments, cells were imaged before treatment, with the location of each cell saved using the automated stage top. Cells were then either treated with 20 ng/mL of IL-6 or 100 nM of insulin and automatically imaged every 5 min for 30 min following treatment.

### 2.6. Fluorescent Image Analysis

All images were processed using FIJI image analysis software [55]. Images were thresholded to isolate for the whole-cell fluorescence by using a triangle threshold [56]. To detect fluorescence near the plasma membrane, the cortical region of the cell was isolated. This region encompasses the plasma membrane and any fluorescence coming from within 300 nm from the edge of the cell [55]. This was accomplished by thresholding for the total fluorescence of the cell using the triangle threshold and an edge detection within FIJI to trace the outline of the cell [55]. To then produce a mask that encompassed all of the plasma membrane, while excluding as much intracellular fluorescence as possible, a Gaussian blur with a sigma value of 2.0 was applied to the outline of cell. This produced a mask with a radius of 300 nm, on images taken with our 63× objective [55]. This allowed for the measurement of the total fluorescence content within 300 nm from the edge of the cell, which was considered as “plasma membrane fluorescence”. To measure the intracellular stores, we then took the area of the cell and isolated for local maxima with at least a 2-fold increase in fluorescence relative to average cell fluorescence to indicate an area of increased concentration of Glut4GFP through the Find Maxima function with a prominence value of 200 [55]. These maxima were then measured for their total fluorescence to give an indication as to the total fluorescence of Glut4GFP in internal stores during different stages of imaging. These values were measured for each cell across all time points. The intracellular stores or the cortical (plasma membrane) fluorescence was then divided by the total fluorescence to give a ratio of the amount of fluorescence coming from internal stores or the plasma membrane compared to the whole cell. These ratios were then normalized to time 0 to a value of 1 such to see the ratio metric change in internally stored and plasma-membrane Glut4GFP relative to the whole cell over time [55,56]. For each IL-6 timepoint, twelve cells were measured between 3 plates on separate days and 9 cells were measured for all insulin time points between 2 separate plates on separate days. A workflow for how this was completed can be seen in supplemental materials.

### 2.7. [^3^H]-2-Deoxy-D-Glucose (2DG) Uptake

To assess glucose uptake, the cells were incubated with HBS containing 10 μM [^3^H]2-deoxy-D-glucose for 10 min at room temperature, as established previously by our group [57]. After the 10 min exposure to the radioactive buffer, the cells were washed with ice-cold 0.9% NaCl followed by lysis with 0.05 N NaOH solution. Cell associated radioactivity was measured using liquid scintillation counting (PerkinElmer, USA).

### 2.8. Statistical Analysis

Statistical significance was determined using a one-way ANOVA followed by a Fishers LSD post hoc analysis. To test for normality, a Shapiro-Wilk test was conducted and in cases where data were not normally distributed, data were logarithmically transformed. For fluorescent microscopy data, time points were determined to be significantly different from each other by using a Mann–Whitney U test. Tests were performed on the ratio between intracellular-store fluorescence:whole-cell as well as plasma membrane fluorescence:whole-cell. Data are expressed as means ± SE with significance set at *p* ≤ 0.05.

## 3. Results

### 3.1. Effect of Acute Insulin and IL-6 Treatments on Signaling Proteins in SH-SY5Y Cells

**Figure 1:**
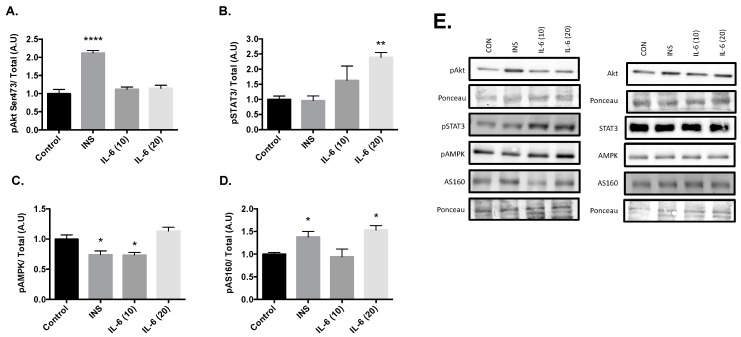
Effects of Acute Insulin and IL-6 Treatment of SH-SY5Y Cells. SH-SY5Y cells were treated with 100nM of insulin, 10ng/mL of IL-6, or 20ng/mL of IL-6 for 30 min. (**A**) Acute insulin treatment significantly increases phosphorylation of Akt at the Serine 473 Site (n = 3 per group). (**B**) Acute IL-6 treatment significantly increases phosphorylation of STAT3 at Tyrosine 705 (n = 3 per group). (**C**) Acute insulin and IL-6 treatment significantly decreases phosphorylation of AMPK (n = 3 per group). (**D**) Acute insulin and IL-6 treatment significantly increases phosphorylation of AS160 (n = 3 per group). (**E**) Representative blots are shown beside the quantified data. Data are presented as means ± SE. A.U., arbitrary units. * *p* ≤ 0.05, ** *p* ≤ 0.01, **** *p* ≤ 0.001, as determined using a one-way ANOVA followed by Fisher’s LSD post hoc analysis.

Cells were stimulated with 100 nM insulin, 10 ng/mL IL-6, or 20 ng/mL IL-6 for 30 min. Post treatment there was an increase in Akt phosphorylation at the Serine 473 site with 100nM insulin compared to the control group (Figure 1A, *p* < 0.001). There were no changes in Akt phosphorylation with either 10 or 20ng/mL Il-6. Significant increases in the phosphorylation of STAT3 at Tyr 705 compared to the control were observed with 20 ng/mL of IL-6 (Figure 1B, *p* = 0.005). However, significant decreases in the phosphorylation of AMPK at Thr 172 compared to the control were observed after treatment with 100nM insulin (*p* = 0.010) and 10ng/mL IL-6 (*p* = 0.014) (Figure 1C). Finally, significant increases in the phosphorylation of AS160 at Thr 642 compared to the control were observed after treatment with 100 nM insulin (*p* = 0.029) and 20 ng/mL IL-6 (*p* = 0.009) (Figure 1D). These results suggest that insulin is working through the Akt pathway, and IL-6 is working through the AMPK pathway. With both insulin and IL-6 significantly activating AS160, it is plausible that IL-6 is capable of promoting GLUT4 translocation, similar to insulin in neurons.

### 3.2. Effect of Acute IL-6 Treatment of SH-SY5Y Cells over Time

**Figure 2:**
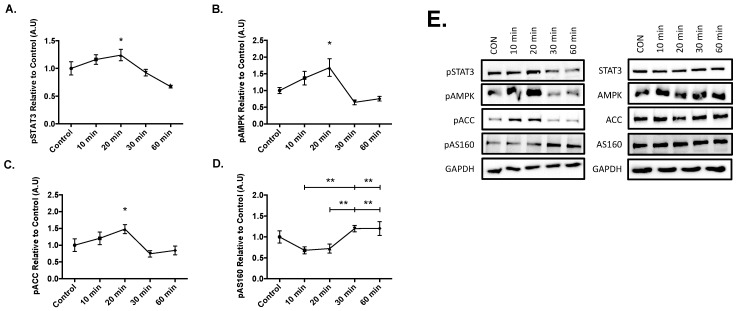
Effect of Acute IL-6 Treatment of SH-SY5Y Cells Over Time. SH-SY5Y cells were treated with 20 ng/mL of IL-6 for 10, 20, 30, and 60 min. (**A**) IL-6 treatment significantly increases phosphorylation of STAT3 (n = 3 per group). (**B**) IL-6 treatment significantly increases phosphorylation of AMPK (n = 3 per group). (**C**) IL-6 treatment significantly increases phosphorylation of ACC (n = 3 per group). (**D**) IL-6 treatment significantly increases phosphorylation of AS160 (n = 3 per group). (**E**) Representative blots are shown beside the quantified data. Data are presented as means ± SE. A.U., arbitrary units. * *p* ≤ 0.05, ** *p* ≤ 0.01, as determined using a one-way ANOVA followed by Fisher’s LSD post hoc analysis.

Results from the time course experiments yielded significant increases in the phosphorylation of STAT3 at Tyr 705 (*p* = 0.050), AMPK at The 172 (*p* = 0.026), and acetyl-coA carboxylase at Ser 79 (ACC, *p* = 0.037) compared to the control after 20 min of 20ng/mL IL-6 treatment (Figure 2). Lastly, significant phosphorylation of AS160 occurred at 30-min when compared to the 10 (*p* = 0.005) and 20-min (*p* = 0.009) time points. Furthermore, significant AS160 phosphorylation also occurred at the 60-min when compared to the 10 (*p* = 0.005) and 20-min (*p* = 0.009) time points (Figure 2D). These results suggest that IL-6 treatment sequentially activates STAT3, AMPK, and ACC before AS160, as AS160 was activated at the later time points in the time course.

### 3.3. GLUT4-GFP Live Cell Fluorescent Imaging

**Figure 3:**
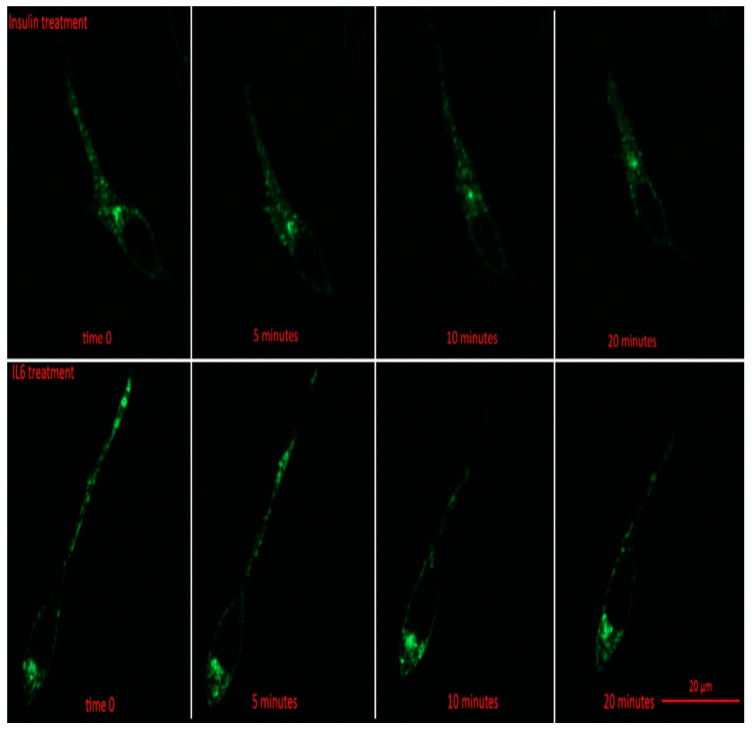
GLUT4-GFP Localization Following IL-6 and Insulin Treatment in Differentiated SH-SY5Y Cells. SH-SY5Y cells were treated with 100 nM of insulin, and 20 ng/mL of IL-6 for 5, 10, 15, 20, and 30 min. Fluorescent images of GLUT4-GFP in SH-SY5Y prior to and 5, 10, and 20 min post treatment with either IL-6 (**bottom**) or insulin (**top**).

**Figure 4:**
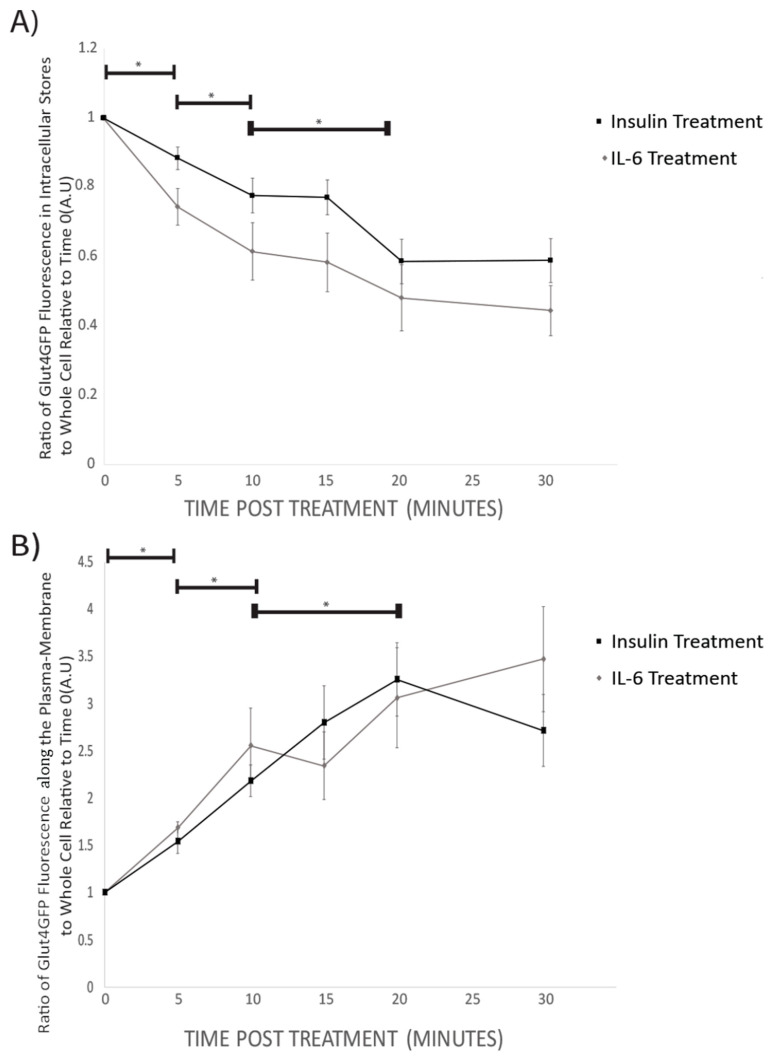
GLUT4-GFP Fluorescent Localization Quantification Following IL-6 and Insulin Treatment in Differentiated SH-SY5Y Cells: SH-SY5Y cells were treated with 100nM of insulin, and 20ng/mL of IL-6 for 5, 10, 15, 20, and 30 min. (**A**) Ratio of intracellular store to whole-cell fluorescence of GLUT4-GFP Decreases with time both in IL-6 (n = 12) and insulin (n = 9) treated cells. (**B**) The ratio of GLUT4GFP along the plasma-membrane to whole cell fluorescence increases with time in both IL-6 (n = 12) and insulin (n = 9) treated cells. Data are presented as means ± SE. A.U., arbitrary units. * *p* < 0.05 as determined using a Mann-Whitney U test between each time point.

Prior to insulin or IL-6 treatment, GLUT4-GFP was found to be mainly in intracellular stores of the cell (Figure 3). Following 5 min, and all subsequent time points for both IL-6 and insulin treatments, total GFP fluorescence from intracellular stores significantly decreased compared to time 0 (*p* < 0.001) (Figure 4A). For both IL-6 and insulin treatments, GLUT4-GFP in intracellular stores significantly decreased further when comparing 5–10 min post-treatment (*p* < 0.018) as well as 10–20 min post-treatment (*p* < 0.045) (Figure 4A).

While intracellular stores fluorescence decreased, there was a significant increase in total GLUT4-GFP fluorescence in the plasma membrane of the cells following 5 min (*p* = 0.042), and all subsequent time points post-IL-6 and -insulin treatment (*p* < 0.001) (Figure 4B). There was a further significant increase in GLUT4-GFP that was localized near the plasma membrane when comparing 5 to 10 min (*p* = 0.024) as well as between 10 and 20 min post-treatment (*p* < 0.031) (Figure 4B). Throughout the experiment, no significant difference between IL-6 and insulin treatments were found at any time point. Results from the imaging experiments suggest that IL-6 treatment promotes GLUT4 subcellular re-localization in a similar fashion to insulin.

### 3.4. Effect of Acute IL-6 on Glucose Transport in SH-SY5Y Cells

**Figure 5:**
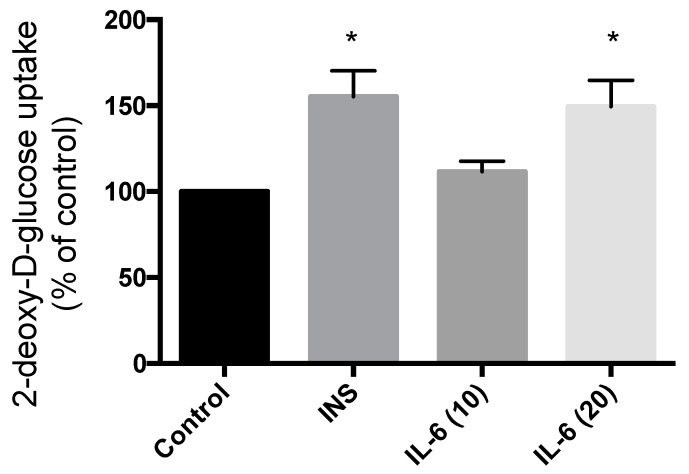
Effect of Acute IL-6 and Insulin Treatment on Neuronal Cell Glucose Uptake. SH-SY5Y cells were treated with 100 nM insulin, 10 ng/mL, or 20 ng/mL IL-6 for 30 min followed by glucose uptake measurements. Acute insulin and IL-6 (20 ng/mL) treatment significantly increase glucose uptake (CON, n = 4; INS, n = 4; IL-6 (10), n = 6; IL-6 (20), n = 6). Data are presented as mean ± SE of 6 independent experiments, expressed as percent of control * *p* ≤ 0.05, as determined using a one-way ANOVA followed by Fisher’s LSD post hoc analysis.

Treatment of SH-SY5Y cells with 100 nM insulin for 30 min resulted in significant increase in glucose uptake (55.2% increase compared to control; *p* = 0.0063) (Figure 5). Exposure to 10 ng/mL IL-6 for 30 min did not have any effect on the glucose uptake (11.6% compared to control; *p* = 0.53) (Figure 5). However, exposure to 20 ng/mL IL-6 for 30 min resulted in a significant increase in glucose uptake to levels comparable to insulin (49.4% increase compared to control; *p* = 0.011) (Figure 5). These data indicate that IL-6 exhibits an insulin-like effect to increase glucose uptake in SH-SY5Y neuroblastoma cells.

## 4. Discussion

The results of the present study are the first to demonstrate the ability of IL-6 to phosphorylate proteins involved in GLUT4 translocation to the plasma membrane in SH-SY5Y neuronal cells. Furthermore, this study is the first to show translocation of GLUT4 vesicles to the neuronal plasma membrane in response to IL-6 treatment as well as increased glucose uptake. These results further demonstrate that there was no difference in neuronal GLUT4 mobilization between IL-6 and insulin, even though they seem to be working through different pathways (AMPK vs. PI3K/Akt). Since individuals with sporadic AD often experience brain insulin insensitivity and hypometabolism, this research is important to help find alternative pathways to increase neuronal glucose uptake in an insulin-independent manner [6,8,58].

Sporadic AD shares a similar metabolic profile to T2DM, which has leaded some researchers to name this disease “Type 3 Diabetes” [27,28]. Some specific characteristics that sporadic AD shares with T2DM are insulin resistance, decreased glucose transport and an overall glucose hypometabolism [59]. Therefore, research to help find treatments for sporadic AD may benefit from treating the disease as a diabetes-like disease. It is known that insulin-dependent glucose uptake occurs through the PI3K/Akt pathway in several metabolic tissues, including the brain [59]. However, there is also insulin-independent mechanisms for glucose uptake, such as activation of AMPK. AMPK is activated by phosphorylation of the Thr172 site and can then directly phosphorylate AS160, which directly interacts with the GSVs [46,47]. The interaction of phosphorylated AS160 and the GSVs promotes GLUT4 translocation to the plasma membrane to allow for glucose uptake into the cell [46,47].

In this study, IL-6 treatment resulted in AMPK phosphorylation, GLUT4 translocation, and increased glucose uptake, which is a similar to the mechanism already characterized in skeletal muscle [46,47]. AMPK is an important regulator of cellular energy and metabolism and is activated in response to an increase in the AMP-to-ATP ratio [60]. Stresses that deplete cellular ATP supplies, such as low glucose, have been shown to activate this protein [60]. Specifically, in the brain there has been new research that shows GLUT4 is present in nerve terminals and neuronal activity can stimulate the recruitment of these vesicles to the presynatptic plasma membrane [24]. Furthermore, there is strong evidence to suggest GLUT4 vesicles have an essential role for synaptic function during prolonged activity, and AMPK is responsible for recruiting these vesicles during the activity [24]. This evidence supports the findings of the present study that showed acute IL-6 treatment resulting in subsequent AMPK phosphorylation and GLUT4 mobilization to the neuronal plasma membrane along with increased glucose uptake. Activation of AMPK could be one potential solution to regulate outcomes seen with sporadic AD, due to IL-6 signalling proteins working through an insulin-independent mechanism [59]. Moreover, because IL-6 and insulin work through different signalling cascades future research could look at using insulin and IL-6 additively to improve glucose homeostasis and metabolism. There is already evidence in L6 skeletal muscle cells that IL-6 can have an additive effect on insulin-stimulated glucose uptake as well as GLUT4 translocation to the membrane [50]. This could be due to the fact that both of these proteins elicit their responses through different signaling pathways. Furthermore, it is known that decreased AMPK activation in skeletal muscle results in the development of insulin resistance and decreased glucose transport [46,47]. That being said, normal AMPK activation in skeletal has been demonstrated to have many diverse beneficial effects; one in particular is its ability to re-sensitize insulin and subsequent glucose transport [61]. Therefore, it can be hypothesized that AMPK activation in the brain could lead to increased insulin sensitivity overtime, which is especially important for individuals with type 3 diabetes.

There is evidence to suggest the brain secretes IL-6, and can subsequently pass through the blood brain barrier, in an acute manner post exercise for a reason that is not completely understood [62]. This study showed that acute IL-6 exposure to neurons results in GLUT4 translocation and can potentially help regulate glucose uptake through an insulin-independent mechanism. Therefore, exercise can be considered a helpful modality for increasing acute IL-6 secretion, which may be especially beneficial for individuals with sporadic AD. Moreover, IL-6 has also been shown to have a role in the brain, specifically in regards to synaptic formation, cell survival, healthy CNS development/maintenance and is also present in healthy aging populations [63,64]. This study highlights the potential role acute IL-6 plays in regulating AMPK activation and GLUT4 translocation, which could contribute to improved synaptic formation, cell survival, and healthy CNS development/maintenance.

The neuronal cell line used in the current study is a well-established cell line used for neuronal research [51], however, it is possible that differentiated SH-SY5Y cells may not reproduce all neuronal features and the use of primary neuronal culture may provide additional information. However, most commercially available primary neuronal cell lines are established from neonates and may behave differently than adult neuronal cells. The use of primary mammalian neurons derived from embryonic central nervous system tissue is limited by the fact that once terminally differentiated into mature neurons, the cells can no longer be propagated. In the present study, using the SH-SY5Y cells, we gained important new information about the basic mechanistic outcomes of acute IL-6 treatment and we recognize that future studies are required to examine the role of IL-6 signalling in primary neuronal cultures from aged mice.

## 5. Conclusions

To conclude, our study is the first to show that IL-6 treatment results in the phosphorylation/activation of STAT3, AMPK, ACC, AS160, and increased glucose uptake in SH-SY5Y neuronal cells independently of insulin. Furthermore, GLUT4 vesicles translocated to the plasma membrane in neurons as a result of IL-6 treatment, similar to insulin. This experiment provides mechanistic insight into a potential role for IL-6, specifically regarding how glucose uptake in the brain can be regulated, and will set the foundation for future studies. Together, this information will significantly increase our fundamental understanding of the processes that underlie how brain glucose metabolism is regulated.
